# 慢性淋巴细胞白血病微小残留病检测与临床解读中国专家共识（2023年版）

**DOI:** 10.3760/cma.j.issn.0253-2727.2023.03.002

**Published:** 2023-03

**Authors:** 中国抗癌协会血液肿瘤专业委员会 中华医学会血液学分会 中国慢性淋巴细胞白血病工作组

慢性淋巴细胞白血病（CLL）是一种CD5^+^CD19^+^的成熟小B细胞在外周血、淋巴组织器官、骨髓积聚的慢性淋巴增殖性疾病，老年人多见。化学免疫治疗（chemoimmunotherapy，CIT）仍是预后良好且可耐受的CLL患者的治疗选择之一，布鲁顿酪氨酸激酶抑制剂（BTKi）、磷脂酰肌醇3激酶抑制剂（PI3Ki）及B细胞淋巴瘤2（BCL2）抑制剂（BCL2i）等新型靶向药物使CLL的治疗进入无化疗时代，显著改善了患者的预后[1]–[4]。近年来，BTKi联合CIT、BTKi联合BCL2i±CD20单抗、BCL2i联合CD20单抗等新的固定周期疗法是临床研究的热点且部分已被批准应用于临床，传统的疗效评估很难精确预测患者的复发及长期生存，而缓解深度是影响患者生存的最重要因素之一。微小残留病（MRD）是导致疾病复发的根源，其不仅与无进展生存（PFS）及总生存（OS）密切相关，也能准确反映治疗后的肿瘤负荷、有效地评估缓解深度，因此已成为治疗终点的重要判定标准[1],[5]–[7]。为推动CLL患者MRD检测和应用，中国抗癌协会血液肿瘤专业委员会、中华医学会血液学分会和中国慢性淋巴细胞白血病工作组组织相关专家制定了慢性淋巴细胞白血病微小残留病检测与临床解读中国专家共识。

一、CLL-MRD的定义

CLL治疗后即使达到完全缓解（CR）也不意味着疾病治愈，采用更为先进的诊断技术检测出外周血（PB）或骨髓（BM）中极低水平的CLL细胞，称为CLL-MRD。患者体内MRD阴性可能存在两种情况，一是确实已经不存在CLL细胞，二是由于检测敏感性限制导致无法检测到微量CLL细胞。因此，有专家认为用可检测到的残留病（measurable residual disease）来表述MRD更为客观精确[8]。欧洲CLL研究倡议组织（ERIC）和国际慢性淋巴细胞白血病工作组（iwCLL）将10^−4^作为临床判断CLL-MRD阴、阳性的阈值。残留的CLL细胞低于万分之一个白细胞（<10^−4^）被定义为uMRD（undetectable MRD），以可检测（detectable, d）或不可检测（undectable, u）进行阈值区分，以MRD4、MRD5表明残留病上限，如MRD4代表残留病灶<0.01％或10^−4^。

二、CLL-MRD检测的方法

目前用于CLL-MRD的检测方法有多参数流式细胞术（multiparametric flow cytometry, MFC）、二代测序（next generation sequencing, NGS）等[8]。常见方法及比较见表1。

**表1:** 不同方法检测CLL-MRD的比较

方法	MFC	ASO-PCR	NGS
敏感性	10^−4^～10^−5^	10^−4^～10^−5^	10^−5^
检测方法	采用不同抗体组合检测细胞表面抗原	PCR特异性扩增患者CLL IgH序列	使用引物扩增所有IgH基因片段后检测CLL特异性IgH序列
是否常规开展	是	是	否
新鲜样本	是（<72 h）	否	否
完成时间	1 d	3 d	1周
前瞻性临床试验中独立预后因素	PFS和OS	PFS和OS	评估中
影响因素	正常B细胞干扰、抗体质量、分析人员的工作经验等	需要基线样本、靶基因丢失、耗时、假阳性率较高	需要基线样本、费用高、耗时、未标准化

注 CLL：慢性淋巴细胞白血病；MRD：微小残留病；MFC：多参数流式细胞术；ASO-PCR：等位基因特异性聚合酶链反应；NGS：二代测序；IgH：免疫球蛋白重链；PFS：无进展生存；OS：总生存

（一）CLL-MRD检测样本的选择

检测样本为PB或BM。PB与BM结果的一致性受治疗方案、治疗时间及病灶空间异质性的影响。PB的MRD检出率往往低于BM[9]。为减少有创性操作、节省检测费用，推荐先做PB，如MRD阴性再做BM。对于旨在实现深度缓解的治疗，推荐同时检测PB和BM[10]。由于淋巴结、肝、脾等部位可能有残留病，NGS技术检测血浆游离肿瘤DNA可能弥补检测PB或BM的不足，减少空间异质性带来的差异。MFC检测样本推荐以肝素或乙二胺四乙酸盐（EDTA）抗凝，72 h内完成[11]；BM样本推荐涂片后的第1管2 ml左右，以避免抽取过程中PB稀释。NGS检测样本推荐EDTA抗凝，新鲜样本优先，−80 °C及以下冻存样本也可使用，样本量要求初诊时1～3 ml，随访时3～5 ml以保证DNA提取量满足检测下限[12]。

（二）CLL-MRD的MFC方法

MFC是目前最常用的检测方法，近年来八色及以上单管抗体组合的应用降低了多管检测因细胞分布不均所导致的结果偏差[9],[13]–[14]，具有更高的敏感性和特异性。

1. CLL免疫表型：MFC检测CLL-MRD的依据是通过细胞表面一系列抗原的特征性表达识别CLL细胞。典型的CLL免疫表型为：CD19^＋^、CD5^＋^、CD23^＋^、CD10^−^、FMC7^−^、CD81^−^、CD43^+^、ROR1^＋^、CD200^＋＋^、CD45^＋^；表面免疫球蛋白（sIg）、CD20、CD22和（或）CD79b通常表达减弱或缺失，细胞表面限制性表达κ或λ轻链以确认B细胞克隆性[15]–[16]。判断某个抗原的表达强度应以对应阶段的正常B细胞作为内参照，如平均荧光强度（mean fluorescence intensity，MFI）高于正常B细胞判断为强表达，反之则为弱表达。

2. MFC检测CLL-MRD的抗体选择：CLL-MRD选用的抗体应尽可能简化且能够充分区分正常成熟B细胞和CLL细胞。专家组结合国内外报道及国内各大实验室的经验，对CLL细胞高频异常表达且在治疗前后表达较稳定的抗原进行筛选，推荐了八色抗体组合方案，包括CD19、CD20、CD79b、CD5、CD81、CD43、ROR1和CD45，除CD45外，其他7个抗原在CLL细胞中的表达均具有特征性，为CLL-MRD检测的核心抗体组合[9],[11]。另外，受样本储存时间、处理制备过程和细胞碎片的影响，仅凭FSC/SSC散射光特点设门圈出有核细胞作为计算MRD的分母，很难保证结果的准确性和一致性，因此，在此抗体组合上增加CD45设门。通过CD45设门圈出白细胞后，再分析7个核心抗原的表达特征，以所有白细胞数作为计算MRD的分母可以有效提高不同实验室结果判读的一致性和结果的准确性，这在多中心临床药物试验的比较中尤为重要。如果实验室没有八色及以上仪器，可参照ERIC推荐的四色或六色方案进行[13]–[14]。

目前国际上通用的可重复识别MRD细胞群体的最低检出限（limit of detection，LOD）为20个成簇细胞，即LOD＝20/获取的白细胞总数×100％[13],[17]。据此，至少需要获取20万个白细胞，灵敏度才能达到10^−4^。而通用的识别MRD细胞群体的最低定量限（limit of quantitation, LOQ）为50个成簇的细胞，即LOQ＝50/获取的白细胞总数×100％。

3. MFC设门策略及分析步骤：详见图1。分析时应注意患者接受抗CD20单抗治疗后可能导致CD20表达减弱甚至消失。此外，圈定总B细胞的设门抗体不能仅依赖CD19，接受CD19-嵌合抗原受体（CAR）-T细胞治疗后的患者可采用ROR1联合CD24作为替代设门标记[18]。

**图1:**
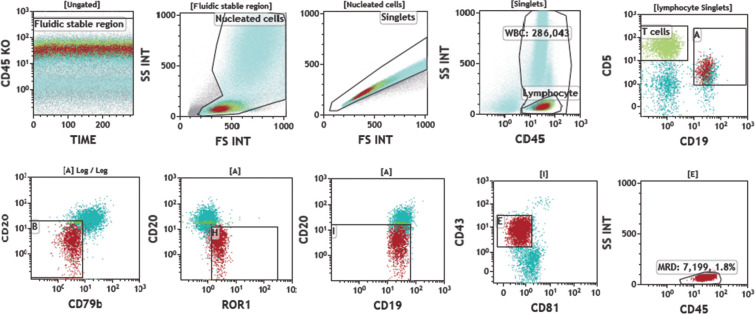
多参数流式细胞术检测微小残留病（MRD）分析流程图 注 利用Time/CD45，FS-INT/PEAK和FS INT/SS INT散点图，圈出液流稳定区间、去除黏连体和细胞碎片；通过CD45/SS圈出所有白细胞群和淋巴细胞群；设门圈出CD19^+^B淋巴细胞（A门）和CD5^+^T淋巴细胞；B-E门：圈出CD20^−/dim^CD79b^−/dim^CD5^+^CD19^+^CD43^+^ROR1^+^CD81^−^CD45^+^细胞群，即为CLL-MRD细胞（红色），计算MRD结果用最终确定的CLL细胞数（7 199个）除以白细胞总数（286 043个），即MRD占白细胞的2.52％

免疫球蛋白轻链κ或λ限制性表达是判断包括CLL在内的成熟B细胞淋巴瘤单克隆的重要标记。由于近1/4的CLL患者不表达轻链，且轻链检测受样本溶血、凝集及制备中裂解红细胞步骤影响较大，因此不建议在CLL-MRD检测方案中加入κ和λ[9],[14]。而ROR1在几乎所有CLL中高表达，因此，专家组推荐的八色方案适用于绝大多数的CLL患者。对于极少数ROR1阴性同时免疫表型不典型的CLL，可增加κ和λ协助判断。虽然10^−4^能满足临床常规疗效评估需要，但对于追求疾病缓解深度最大化的临床试验和CAR-T细胞或造血干细胞移植等治疗手段，选择10^−5^～10^−6^作为阈值可能更具临床价值。通过MFC获取细胞数达2×10^6^时，敏感性能到10^−5^以上，称之为二代流式细胞术（NGF）。因其需要更多的样本量、抗体、检测时间和费用，对MRD敏感性要求高的实验室可选择开展[19]。

4. MFC检测的CLL-MRD报告方式：报告应涵盖以下内容：①患者基本信息及标本来源部位、时间，标本是否有凝块、溶血、特殊原因未能及时检测等情况；②罗列出检测所用的所有抗体及荧光素、仪器型号、样本制备和红细胞裂解方法；③获取的白细胞总数，同时报告方法学LOD；④如果没有检测到确定的CLL-MRD细胞群体（<20个），在报告上写明MRD阴性，或未检测到；如MRD阳性，应根据MRD细胞数占白细胞的百分比报告，同时描述该群细胞的免疫表型特点。例如，在20万个白细胞中，检测到CLL细胞低于20个，报告：未检测到CLL-MRD；检测到60个CLL细胞，报告：CLL细胞占所有白细胞的0.03％（LOQ：0.025％）；检测到30个CLL细胞，报告：检测到CLL-MRD，低于LOQ（LOQ：0.025％）。

5. 质量控制：建议参照《多参数流式细胞术检测急性白血病及浆细胞肿瘤微小残留病中国专家共识（2017年版）》相关内容[17]。

（三）CLL-MRD的分子生物学检测方法

分子学检测方法主要包括实时定量PCR技术（qPCR）与NGS[8]。CLL免疫球蛋白基因重链（IgH）的互补决定区3（CDR3）区域VDJ重排模式具有细胞特异性标记且保持不变，是CLL-MRD检测的理想分子标志物。通过确定初诊时IgH克隆性重排模式与使用片段基因序列信息，在后续随访中以此进行动态监测[20]。等位基因特异性核苷酸探针法（ASO）RQ-PCR为最具代表性的RQ-PCR技术，灵敏度可达10^−5^[21]；NGS可分别基于DNA及RNA样本，采用cloneSEQ和RNA-seq平台进行检测，灵敏度可达10^−6^[22]–[23]。专家组推荐常规开展MFC检测CLL-MRD，有条件的中心开展NGS。

综合考虑样本可及性及实验结果稳定性，现NGS开展的CLL-MRD检测主要基于DNA样本，流程包括：①采用扩增子法制备文库并精确定量，通过系列引物采用多重PCR法扩增DNA目的区域片段；②根据目标产物长度、检测通量、所需数据量等因素选择合适的NGS测序仪（如Illumina、MGI、Thermo Fisher等）上机测序；③对测序数据进行生物信息学分析：包括生信质控、数据过滤、序列比对、加入内参序列并矫正扩增效率，对克隆序列拷贝数进行绝对定量、假阴性及假阳性质控等流程。

分子生物学MRD结果的报告应涵盖以下内容：①患者基本信息及样本类型、采集时间，检测技术及敏感性，结果解释说明；②初诊样本报告列出优势克隆性重排类型、序列及其占比；③MRD监测报告需列出优势克隆性重排对应检测结果，动态监测应列出每次检测时间点并绘制动态变化曲线[22],[24]。

三、CLL-MRD检测的临床解读

（一）检测时机的选择

推荐治疗过程中用同一方法监测：①对于CIT及新的固定周期治疗，推荐与疗效评估同时进行，末次治疗结束后至少2个月进行；②接受持续治疗的患者，可在获得最佳临床疗效时进行，临床试验中推荐按照研究设计在固定时间点监测；③BTKi单药或联合CD20单抗治疗缓解深度有限，不常规开展MRD检测，在疾病稳定或缓解程度不断加深的患者中可每年定期检测；④部分缓解（PR）患者也可获得uMRD，因此MRD评估不应局限于CR患者[25]。

（二）MRD优化疗效评估和预后的应用价值

目前CLL的疗效评估主要参考iwCLL标准，评价肿瘤负荷和骨髓造血功能。CLL-MRD反映残留病负荷，是临床结局的独立预后因素。①MRD可作为疗效评估和反应缓解深度的补充：CIT治疗结束后CR伴uMRD与PR伴uMRD患者PFS的差异无统计学意义，优于CR伴MRD阳性患者[25]；而PR伴uMRD患者中，存在残留淋巴结病灶患者的PFS较脾脏肿大患者差。一线及复发难治CLL患者接受BCL2i±CD20单抗治疗后，获得uMRD的PR与uMRD的CR患者PFS差异无统计学意义。②MRD与PFS的关系：治疗结束后（EOT）的MRD状态已在CIT时代不同年龄及强度的一线治疗方案下证实可独立预测PFS[25]，BCL2i±CD20单抗治疗后的MRD水平也可预测PFS。异基因造血干细胞移植后1年，以10^−6^为阈值的uMRD也与远期无复发相关[26]。接受BTKi±CD20单抗治疗的患者获得的缓解深度有限而可达到长期PFS，MRD不适合作为PFS的替代指标。③治疗结束后不同水平的MRD对PFS的影响：CIT及固定周期的BCL2i±CD20单抗治疗下，MRD<10^−4^（即阴性）患者的PFS时间较MRD在10^−4^～10^−2^及MRD≥10^−2^患者延长[27]–[28]，MRD<10^−6^的患者PFS时间更长；而有研究显示，MRD在10^−5^～10^−6^的患者的PFS与MRD<10^−6^的患者相比无明显差异[29]–[30]。此外，不同治疗下影响MRD的因素不同，治疗线数、IGHV突变状态、TP53突变、del（17p）是CIT治疗下的影响因素[8],[31]；而BCL2i±CD20单抗治疗下的影响因素包括TP53突变、del（17p）、复杂核型等[28],[30]。因此，欧洲药品管理局（EMA）目前批准MRD在临床试验中作为PFS的替代终点，而不作为临床常规中PFS的替代。专家组推荐在不同治疗模式的临床实践及临床试验中，结合iwCLL疗效标准、MRD、PFS及OS进行个体化及综合评估，研究影响MRD的因素及其对PFS的影响。

（三）动态监测MRD的应用价值

在不同治疗模式下，在治疗期间及随访中动态监测MRD，其应用价值在于：①MRD作为指导治疗的依据：既往CIT治疗下，3个疗程FCR方案后MRD≤1％提示EOT获得uMRD可能性高，PFS获益，3个疗程后uMRD的患者即使不接受额外疗程，对PFS无影响[32]；BTKi联合CIT、BTKi联合BCL2i±CD20单抗等固定周期治疗中，目前常以固定时间点的骨髓uMRD作为主要或次要观察终点，参考用于后续治疗的选择，或确定治疗时长；而根据早期MRD下降速度及深度，有望识别出通过短期联合可实现停药的患者群体[10],[33]–[34]。②MRD增长动力学探索：不同治疗模式下MRD增长动力学不同，BTKi联合BCL2i±CD20单抗治疗下uMRD获得率及停药后维持率均较高，停药后MRD增长速度较CIT慢。影响MRD增长的因素包括治疗方式、治疗前肿瘤负荷、EOT的BM-MRD水平、慢性淋巴细胞白血病国际预后指数等[28],[30]。③MRD复发概念尚未明确，目前认为需至少连续2次PB MRD>10^−4^为MRD复发，其可作为亚临床进展的标志物；但MRD动态监测时间间隔、MRD复发与临床复发的关系、是否可潜在指导治疗调整等，仍需临床试验探索。④MRD的克隆演变模式探索：在固定疗程BCL2i联合抗CD20单抗治疗结束后达到uMRD的患者中，合并del（17p）、复杂核型、IGHV无突变者在停药后进展概率大[28]。动态监测MRD有利于探索在不同治疗模式下如TP53突变、NOTCH1突变等高危因素及BTK、PLCG2、BCL2等获得性基因突变的克隆演变规律；固定疗程的BTKi联合BCL2i治疗结束停药的患者中，长期随访疾病进展时无耐药突变，再启动原治疗有效，间接提示在CLL中实现“治疗假期（holiday）”。因此，推荐在不同的治疗模式下分析影响MRD动力学的因素，识别真正可能临床治愈及适合固定周期治疗的患者；分析克隆演变规律，减少药物不良反应或治疗压力下的克隆选择；同时在动态监测下进行MRD复发后早期临床干预与等待临床复发的潜在获益等系统研究。

四、小结与展望

总之，随着治疗模式的变化，近年来MFC标准化及分子生物学使CLL-MRD检测取得很大进展。在不同治疗模式下针对不同部位和时间点进行MRD检测，可作为疗效评估的补充、临床试验的观察终点、指导治疗的参考等。未来在以获得深度缓解及停药为目标的临床试验中，可探索MRD的动力学及克隆演变模式。随着MRD研究和临床实践的进展，本共识将不断更新完善。
